# Socialistic Theories in Their Medical Aspect

**Published:** 1855-11

**Authors:** 


### EDITORIAL DEPARTMENT.

Will soon be published, from the press of Messrs. Blanchard & Lea, of Philadelphia, "The Principles and Practice of Physical Exploration, as applied to the Diagnosis of Diseases of the Respiratory Organs. By Austin Flint, M. D."

*Octavo of from* 400 *to* 500 *pages.*

The well known ability of the writer is a sufficient guarantee that this work will be a valuable contribution to that department of medical research. We hope that it may meet with a warm reception among the author's old friends, the subscribers of the Buffalo Medical Journal.

---

*Socialistic Theories in their Medical Aspect.*—The advent of a new novel is hardly a matter for comment in the pages of a medical monthly, and this consideration has induced us to forego an intention we had deliberately formed, of writing, for the pages of the Journal, a full review of "Mary Lyndon," a new novel just published from that fountain of all indecency, the press of Fowler & Co., Nassau-st., New York.

But, in thus abandoning a well considered purpose, we have been actuated rather by the fact that the New York Daily Times has given this book of infamy the benefit of a review, which sufficiently exhibits its moral tendencies, and which has probably met the eye of the larger portion of our readers.

The authorship of Mary Lyndon is avowed by Mrs. Mary Gove Nichols, *M. D.*, one of that crew of belligerent sisters who have invaded our profession, and gone out to the world from the Female Medical Colleges recently established. Connecting herself in a half-wav matrimonial manner with Dr. T. D. Nichols, one of the elite of the Water Cure fraternity, she has taken to the practice of medicine, eking out her income by writing sisterly advice upon the generative organs, and finally by issuing this book, Mary Lyndon.

We can hardly be wrong in supposing that the medical profession has an immediate interest in this discussion. The warmest and most influential advocates of these new revelations in social philosophy write their names with the suffix of M. D., obtained in some of those piratical institutions newly chartered by stultified legislatures, as Eclectic, Homœopathic, or Hydropathic Colleges. One of their favorite methods of teaching is by infusing their moral poison into pretended physiological works. Marriage as a divine ordinance, is especially their aversion. They openly contend that, a marriage becoming distasteful to either of the parties, it is the duty of that party to dissolve the connection, and enter upon a new one with some more congenial spirit. In "Mary Lyndon," Mrs. Nichols, in her medical capacity, asserts that the offspring of unhappy marriages are physiologically imperfect, liable to early death, or, living, to become the prey of disease and passion. Not only this, but we are taught that such a marriage entails ill health and suffering on the parties themselves. From this stand-point an attack is made—not on hasty, ill-considered, or unnatural matches—but on the institution itself, on the Bible as recommending it, and on the legitimate profession of medicine as the well-known opponent of promiscuous intercourse.

In this our profession shares with all other established institutions. In religion, our new-lights vary from the foggy pantheism of Swedenborg, to the gross communism of the Prophet Brigham Young, or the atheism of the Owen's and Fanny Wright.

In politics they are uniformly Socialists in one or another form; that of Individualism, as advocated in the Free Love doctrines of Mrs. Nichols and the Ceresco Union, where each individual must protect his own individual happiness (the logical inference from which is, that as women have no individuality in the marriage relation, that relation is in itself a wrong) or as pure communists or Fourierites, teaching the "higher harmony" of bestial promiscuous intercourse. Such are the doctrines of the New York Tribune—not openly proclaimed, but logically necessary to its theories.

In medicine we find them asserting their natural affinities. The conservative press of our country is firm ini ts advocacy of legitimate medicine; but wherever we find the advocacy of any of these latter-day doctrines, there do we also find a constant and consistent depreciation of our profession. At the risk of startling the prejudices of some of our readers who have not studied this subject, we shall individualize the New York Tribune as a source of more evil to the profession, and support to quackery, than any or all the attacks of our open enemies. Never losing an opportunity to traduce the fame of the true physician,* it has ever been the organ of quackery. The advocate of spiritualism, mesmerism, Fourierism, Free Love, and especially

* Our readers will recollect its malignant attack upon the medical advisers of President Taylor in his last illness.

of the hydropathic and homœopathic delusions, it has never stickled to puff any other form of humbug. For a time it was the mouthpiece of chrono-thermalism, and, inconsistent to the last degree in its advocacy of antagonistic theories, it has been only consistent in its undisguised attacks on the true physician.

We believe that we are right in asserting that a gross sensualism is one of the prevailing characteristics of quackery in its modern form; educated to a refinement that gilds rascality to a scanty semblance of love for the race. We have learned to look upon no man or woman as a thorough, irredeemable, and totally depraved quack, until he has written a book on "Matrimony," or "Love, Courtship and Marriage." All of this pestiferous brood of books inculcate doctrines analagous to those of the Ceresco Union. We could give — did we dare — the private histories of their authors and authoresses, to a large extent — histories of profligacy such as would cause shame itself to blush. Looking, however, among the irregulars, we recognize Mr. L. N. Fowler, author of books which prurient boys keep hid in barns, prudently concealed from the inspection of their parents; Mrs. Joel Shew, author of "Water Cure for the Ladies," of whom we might say not a little; Dr. Lazarus, author of "Passional Hygiene," "Comparative Pschycology," and "Love versus Marriage;"* T. L. Nichols, editor of a smutty paper in New York, superintendent of a Water Cure, author of "Esoteric Authropology" and "Woman in all Ages," and, finally, husband of Mrs. Mary Gove Nichols, a she-professor in some female medical school, author of "Marriage," and who has recently attained to the bad eminence of writing "Mary Lyndon;" Henry C. Wright, a lecturer on the congenial subjects of Hydropathy and Woman's Rights, who utterly repudiates the marriage bond; Mrs. Love, (what a satire in the name!) a prominent sister in that precious band who meet in convention at Rochester and Syracuse to discuss Woman's Rights and Spiritualism, with a petticoat doctor from Boston as their President—ess, and who has recently swindled a divorce from her husband through the courts, that she might connect herself — we will not call it marriage — with Andrew Jackson Davis, the Poughkeepsie Seer, himself author of some remarkable medical works — a name with which we may fittingly close our lengthy list of advocates of MEDICAL REFORM! What a catalogue of infamy!

---

* Dedicated "To all True Lovers — to the modest and brave of either sex, who believe that God reveals to the instinct of each heart, the laws which he destines it to obey; who fear not to follow the magic clue of charm, but defy the interference of all foreign powers!"

In the so-called medical works of these writers may be found the impersonation of all heresy and schism; of hatred to that medical profession which it cannot bend to its doctrines; of advocacy of water cure, homœopathy, and spiritualism; of a monstrous and spurious physiology, whose insane teachings lead its followers over the ruins of the social fabric, morality, and religion; of a debasing sensualism, which in the name of friendship to the gentler sex, would sink them to concubinage.

What does Free Love mean? Simply that the nominal wife may be at any moment, for any whim, deserted and thrown, with her offspring of shame, upon the charities of a world which never forgives a fallen woman.

Such are the doctrines which have mingled with the therapeutics of Hahnemann and Priessnitz, till they are now inseparable from them. The text-books of homœopathy are filled with stupid transcendentalisms, which befog and prepare the mind for that farrago of nonsense to be found in the writings of the spiritualists and communists; that balderdash about "higher harmonies," "marriage of the affinities," and "passional attraction." The very nosology of Hahnemann makes vice a disease, and "a disposition to lie," or "steal," or "murder," is gravely described as cured by "thirtieth potencies." In this view it would be well to confine the practice of homœopathy to penitentiaries and jails!

However wicked may be the tendencies of the infinitesimal delusion—however great an upsetting of all power to discriminate between right and wrong the mere adoption of such a theory must imply—it is in the ranks of hydropathy that rascality has attained to its utmost development. We have shown that most of the water-cure lecturers, and physicians in charge of cures—a number of whom we have mentioned—are also the shameless teachers of doctrines, so infamous that even the liberal use of language permitted in medical writing is insufficient to describe their enormity; doctrines which would make all women concubines, all property plunder, all religion an irresponsible pantheism, and would constitute the "higher law" of the seared conscience of the adulterer the only guide to right and wrong.

It is to the upholders of this heathen creed that pious citizens commit their wives and daughters when they send them to water cures for restoration. We have heard some talk of revelations of the sins against female virtue and modesty committed within the unanswering walls of these places, but whether they are made or not, is a matter of no moment. We cannot, however, resist the impulse to embody here a quotation from Miss Catherine E. Beecher's Letters on Health, just published by the Harpers. As this is a book especially devoted to the advocacy of water-cure, we shall not be charged with unfairness in the source of our selection:

"In my travels I have met persons of both sexes, of the highest cultivation and refinement, whose conduct was every way reputable, and whose morals were never in any way impeached, who freely advocated the doctrine that there was no true marriage but the union of persons who were in love; that such union needed not legal or religious rites, and that it was those only who were held together by such restraints, who, having ceased to love each other, were guilty of adultery in the only proper sense of the word. I have seen books and papers freely circulated that advocate the same view by the most plausible arguments.

"Then, again, there are articles on physiology circulated freely, that maintain that the exercise of all the functions of body and mind is *necessary to health*, and that no perfectly-developed man or woman is possible, so long as any of the functions and propensities are held in habitual constraint. With these creeds is usually combined an entire want of reverence for the Bible as *authoritative* in teaching truth or regulating morals.

"Let us now suppose the case of a physician, neither better nor worse than the majority of that honorable profession. He has read the writings of the semi-infidel school, till he has lost all reverence for the Bible as *authoritative* in faith or practice. Of course he has no guide left but his own feelings and notions. Then he gradually adopts the above views in physiology and social life, and really believes them to be founded on the *nature of things*, and the intuitive teachings of his own mind. Next he has patients of interesting person and character put under his care, and he very naturally takes the means, which these books and papers in his reach afford, to lead them to adopt *his views of truth and right* on these subjects. Then he daily has all the opportunities indicated. Does any one need more than to hear these facts to know what the not unfrequent results must be?

"I will now state, in the first place, that in *no single instance* did I ever know any wrong transpire in any one of the institutions for health in which I have resided, *during the time of my residence there*. Though I had often heard suggestions and intimations, yet never, by the strictest scrutiny, could I ever learn that there was any just ground for want of entire confidence in the professional honor of any one of the medical gentlemen in whose institutions I have resided. At the same time, all the ladies with whom I conversed were unanimous in the same opinion. For, of course, a contrary opinion would immediately banish every respectable person who held it.

"In regard to the health establishments implicated, only one of them was a *Water Cure*, and that one has come to an end. So that every institution now known to me of this description is, so far as I know, free from any such imputation.

"These things being premised, I would state that, *during the last two years*, facts have been brought to my knowledge of a most shocking nature, and from the most unimpeachable sources. The information relied on was not received at second hand, but from ladies of the highest character and position, and involved narratives of their own hazards and escapes.

"In other cases most mournful histories were given from direct and reliable quarters of the most terrible wrongs perpetrated without any possibility of redress, except by a publicity that would inflict heavier penalties on the victims than on the wrong-doers.

"So numerous were the instances that came to my knowledge *unsought*, and from so many different and unsuspected directions, and these cases involved so many guilty perpetrators, not only of those connected with health establishments but in private practice, that a most difficult and painful responsibility became apparent.

"After extensive consultation as to what should be done, it has been decided that these intimations and an article from a medical source prepared for the purpose, would furnish sufficient warning without any details.

"A terrific feature of these developments has been the *entire helplessness* of my sex, amidst present customs and feelings, as to any *redress* for such wrongs, and the reckless and conscious impunity felt by the wrong-doers on this account. What can a refined, delicate, sensitive woman do when thus insulted? The dreadful fear of *publicity* shuts her lips and restrains every friend. And it would seem, from some of the cases here indicated, as if it was the certainty of this that withdrew restraint, so that the very highest, not only in character but in position, have not escaped. When *such as these* have been thus assailed, who can hope to be safe?

"Another alarming circumstance has been the character of the physicians implicated. After intimate acquaintance with some of them, I was impressed with the belief that they were, at least, men of benevolence and professional honor, while in some cases their conversation and deportment led to the hope that they were persons of consistent piety. Of course the painful inquiry has arisen, how can a woman *ever know* to whom she may safely intrust herself or her child in such painful and peculiar circumstances? No doubt the medical profession embraces multitudes of persons of the highest delicacy, honor, and principle, and those who are in long and close proximity can be sure of their rectitude. But how can *the public* discriminate? Some of these guilty men were receiving patients sent to their care by the *regular* physicians, while the great body of their patients, who had escaped all knowledge of their guilt, were earnest in their representation of their high character."

The statement at the close, that "some of these guilty men received patients from the *regular* (italics *not* our own) physicians," is a gratifying proof that Miss Beecher's charges are not intended for regular physicians, but only for the graceless scamps with whom she has unwittingly associated herself, and whom she still supports by her forcible pen.

Did we lack all proof of the actual commission of crime, we might well appeal to the publications of the keepers of these houses, and ask if they are safe men to be entrusted with the control of female honor? But we have said enough of this.

One thing is proven. Between all the various forms of quackery and those of religious and social infidelity, there is such a bond of affinity that we find the same class of minds attracted by both. Those who daintily tamper with the one, are extremely liable to be entrapped by the other. He who admits the propriety of female physicians, asserts female equality. Believing this, he is bound to concede to the equal female her equal rights, and in doing this, he does away with marriage, for in Christian marriage, ordained by God, there is and can be no equality. *Facilis descensus Averni.* The weak chain of reasoning thus traced, has beguiled many a feeble mind.

The truly just and conservative position of the medical profession proper, was never more forcibly impressed upon us, than by the remark of a friend, that all "medical reformers," so far as his acquaintance—a large one—extended, were men of low morality. The honest seeker after truth in medicine finds field enough for labor in the furtherance of reform in the treatment of disease, to be attained by steadfast study and effort, not by abuse and detraction of those who have labored before him.

Error is never barren. One heresy begets another, until the public mind, pleased with the first of the chain, starts back in horror at beholding the natural results of the theory it had sanctioned. Such, we trust, may be the tendency of the developments of the present day—developments which make it manifest that the especial fondness for medical tinkering, characteristic of the "reformers" of the time, is not so much due to faults in medicine itself, as to great truths which it maintains, and which must be overthrown before the contemplated object of their desires can be reached—that object being the overthrow of the present social system, the abolition of marriage, and the creation of a Utopian Republic, a freedom from all law save the "higher law"—the law of the individual—where selfishness would eventually become the highest charity, where health would be sought in a return to the habits of the beast, and Passional Attraction become, what its advocate, Parke Godwin, calls it, the "pivotal idea" of social life.

